# Association between Food Insecurity and Psychological Distress Among Older Kenyan Women

**DOI:** 10.1093/geroni/igaf122.1905

**Published:** 2025-12-31

**Authors:** Beatrice Oppong, Gabrielle Maloney

**Affiliations:** University of Texas Rio Grande Valley, Oxford, Ohio, United States; Drexel University, Philadelphia, Pennsylvania, United States

## Abstract

**Objective:**

Food insecurity is a major influencing factor of chronic psychological distress among older Kenyan women. However, these women experience food insecurity differently, with various factors influencing how it affects them. The aims of this study were as follows: (1) identify distinct subgroups of food insecurity among older Kenyan women based on indicators of food-related hardships, (2) examine the demographic and socioeconomic factors associated with food insecurity cluster membership, and (3) investigate the relationship between food insecurity cluster membership and psychological distress.

**Method:**

274 older Kenyan women were recruited from four rural and urban areas in the summer of 2022. We collected sociodemographic, socioeconomic (including wealth indicators), global health, psychological health, and food availability data. We conducted a two-step cluster analysis using SPSS to identify potential distinctions among groups. A binary logistic regression was also conducted to identify the demographic characteristics of the clusters.

**Results:**

No significant differences were found in demographic and socioeconomic factors between the “often food insecure” and “sometimes food insecure” clusters. Binary logistic regression results also indicated that a unit increase in material wealth is associated with a decrease in food insecurity among older Kenyan women.

**Conclusion:**

Our study examined psychological distress and food insecurity, two areas that are tremendously impacting older Kenyan women. It shines a light on factors that are affecting the mental and physical well-being of this population.

